# Sporadic Creutzfeldt‐Jakob disease: A case report and review of literature

**DOI:** 10.1002/ccr3.3131

**Published:** 2020-07-16

**Authors:** Rajeev Ojha, Gaurav Nepal, Sujan Jamarkattel, Bikram Prasad Gajurel, Ragesh Karn, Reema Rajbhandari

**Affiliations:** ^1^ Department of Neurology Tribhuvan University Teaching Hospital Kathmandu Nepal; ^2^ Maharajgunj Medical Campus Tribhuvan University Institute of Medicine Kathmandu Nepal; ^3^ Department of Internal Medicine Lincoln Medical and Mental Health Center Bronx NY USA

**Keywords:** bovine spongiform encephalopathy, magnetic resonance imaging, prion, Sporadic Creutzfeldt‐Jakob disease

## Abstract

Creutzfeldt‐Jakob Disease is a rare neurodegenerative disease and earlier diagnosis is usually difficult. Combining clinical features with electroencephalogram, laboratory parameters, and neuroimaging findings will facilitate the diagnosis.

## Graphical abstract

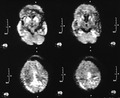

## 1 INTRODUCTION

Creutzfeldt‐Jakob disease (CJD) is a rare and fatal human neurodegenerative disorder characterized by a rapidly progressive dementia, myoclonus, cerebellar, pyramidal, extrapyramidal, visual symptoms, and psychiatric manifestations. CJD occur sporadically in about 85% of cases, 10%‐15% are inherited, <1% are iatrogenic, and <1% are variant.^1^, ^2^ Normal cellular prion protein (PrPC) is found on cell membranes throughout the mammalian body. Disease‐causing form of prion (PrPSc) multiplies by binding to the normal cellular isoform PrP and converts it into an abnormal, structurally altered disease‐causing PrPSc, which then spreads and accumulates throughout the brain leading to spongiform neurodegeneration.^1^ CJD occurs worldwide, but as systematic surveillance has only been undertaken in a minority of countries, the incidence in much of the world is currently unknown.^3^, ^4^ Usually, initial diagnosis of CJD may be obscured by its variable presentation. We herein present a case of a 66‐year‐old female who was admitted to our university hospital for a rapidly progressive cognitive decline followed by ataxia and myoclonus. She was diagnosed with sporadic CJD, based on her clinical features, cerebrospinal fluid (CSF) studies, electroencephalogram (EEG), and brain magnetic resonance imaging (MRI) imaging sequences.

## 2 CASE DESCRIPTION

A 66‐year‐old female was referred to our hospital with 4‐month history of progressive cognitive decline, behavioral, and personality changes. She was in her usual state of health until four months ago when she was noted by her family members to have easy forgetfulness and worsening functional impairment. She was initially noted to be aggressive and stopped taking care of herself. As per the husband, she would have sudden outbursts of agitation and delusional behavior followed by paranoid behavior. She initially presented to a local hospital and was diagnosed with schizoaffective disorder, as no other obvious organic brain lesions were identified. She was subsequently started on antipsychotic medications. A few months later, the patient was noted to have gait disturbances along with dysarthria and ataxia. She continued to worsen with steep functional decline to bedridden stage and became progressively apathetic. She exhibited regressive behavior along with occasional visual hallucinations.

**Figure 1:**
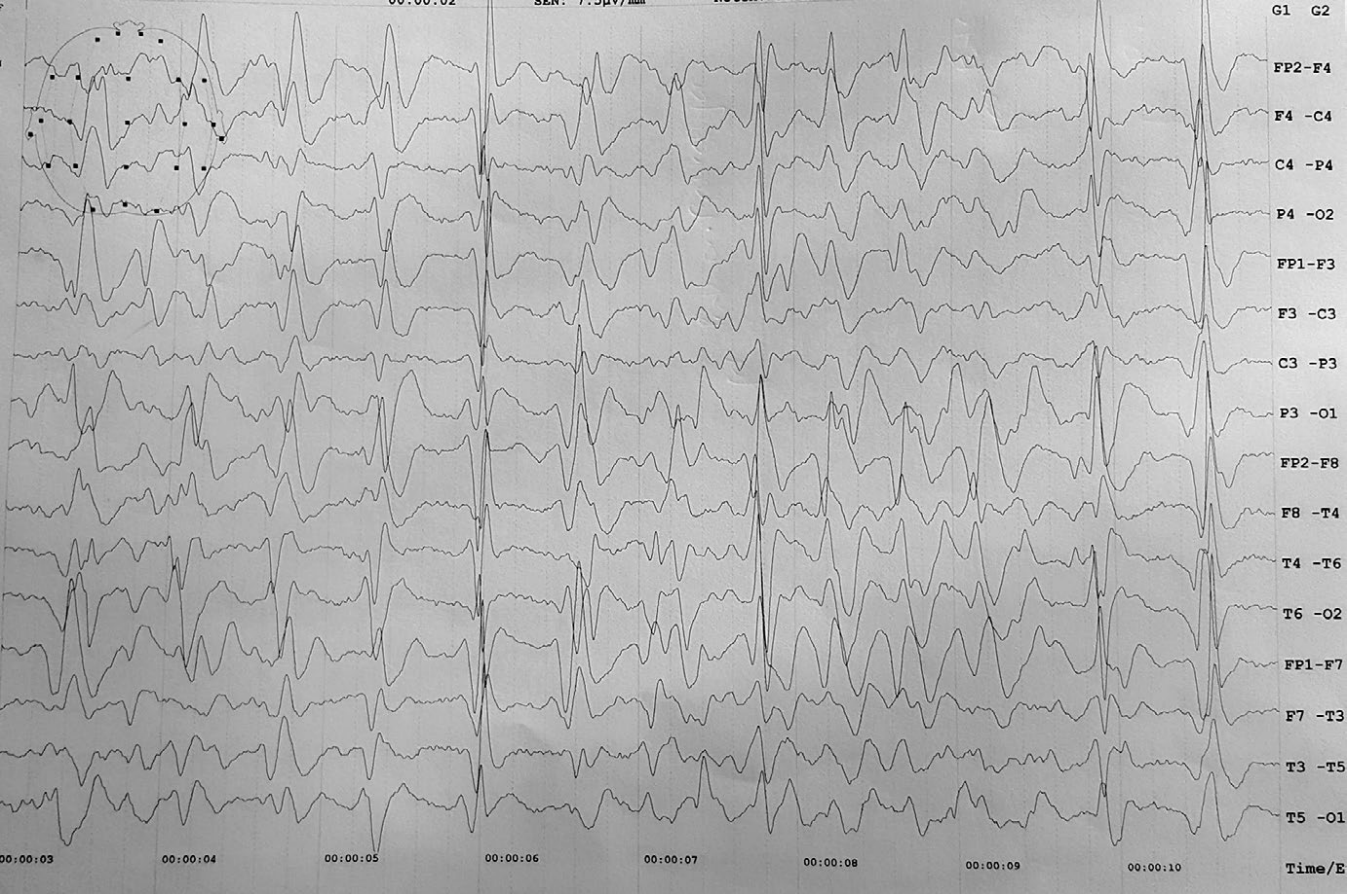
EEG features of continuous periodic complexes and generalized slow waves

**Figure 2:**
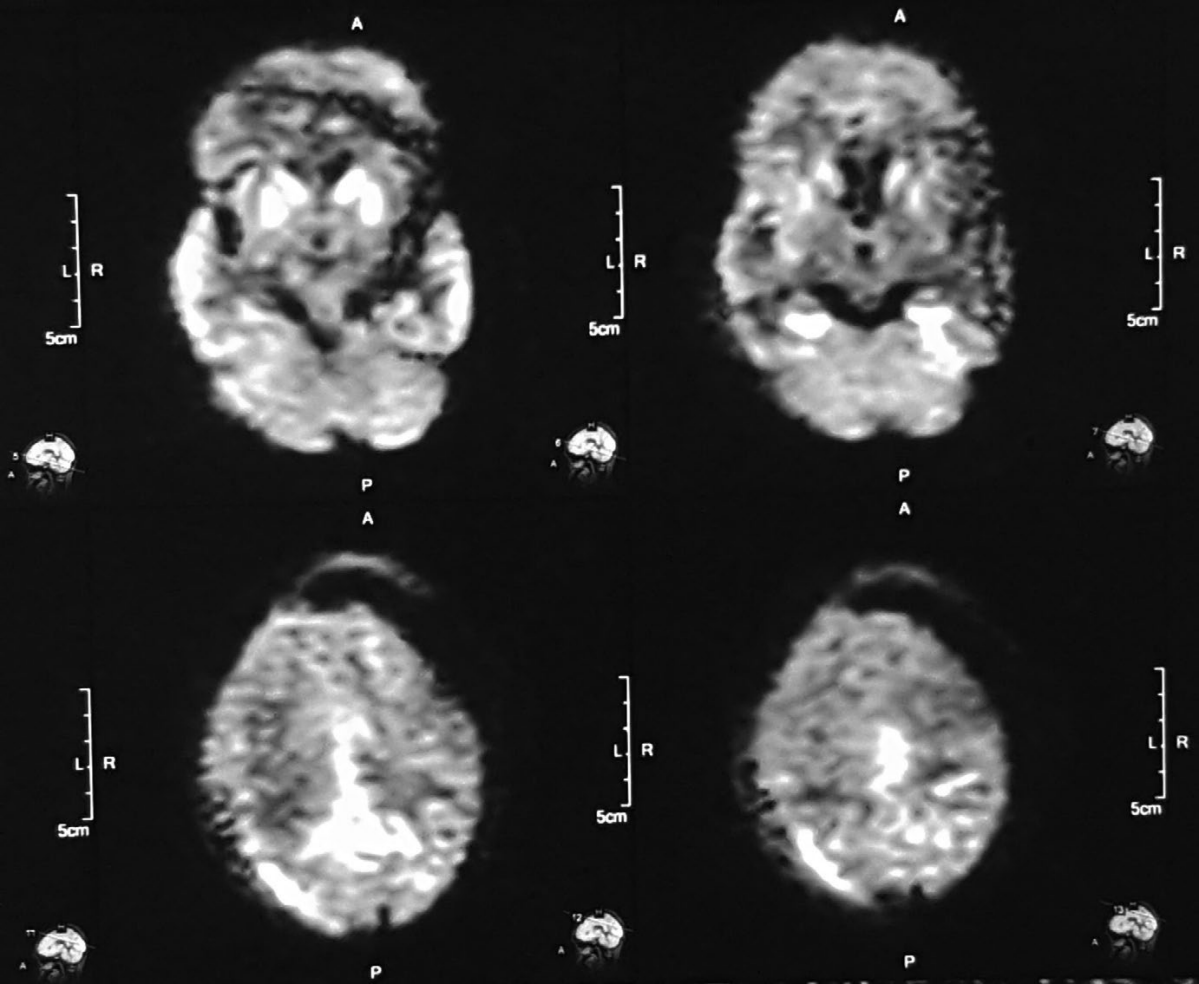
MRI with diffuse‐weighted imaging shows bilateral symmetric basal ganglia hyperintense lesion with cortical ribboning

**Figure 3:**
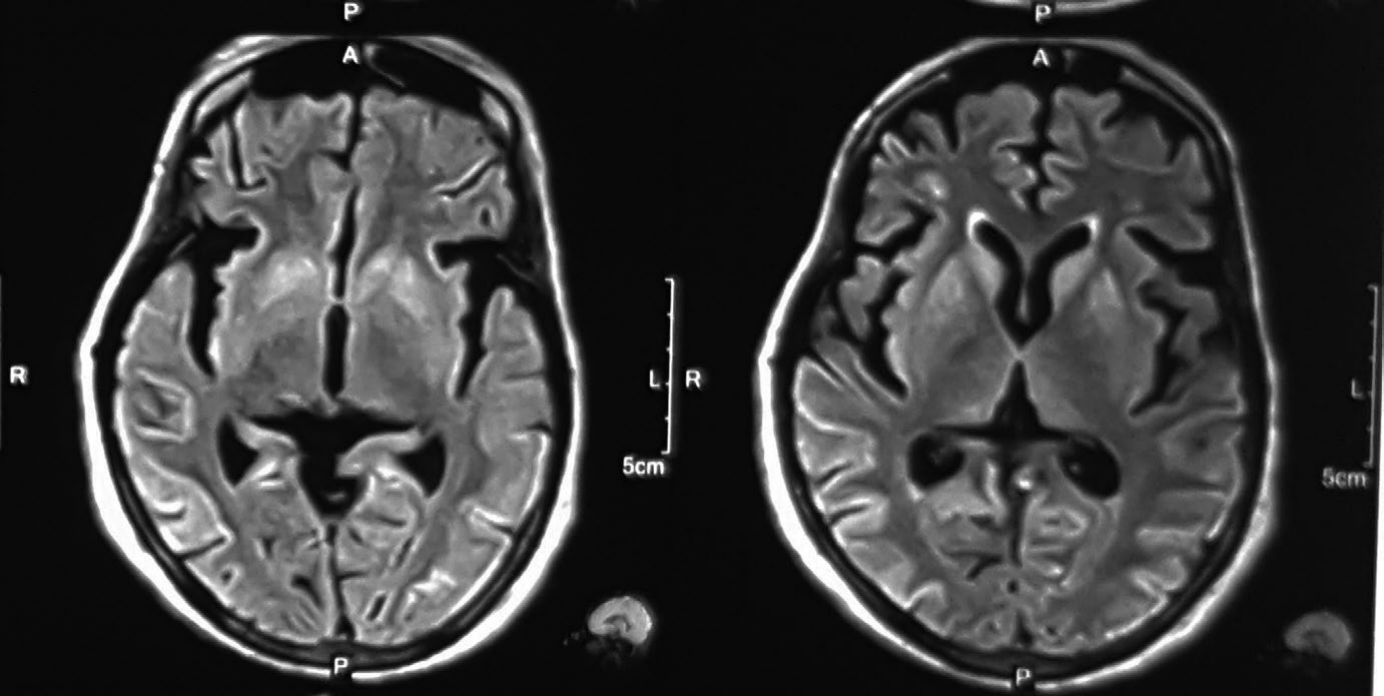
MRI with T2 FLAIR image shows bilateral symmetric basal ganglia hyperintense lesion with cortical ribboning

She was nonsmoker with no significant past medical, surgical, or psychiatric history, and there was no family history of dementia or other neurological disorders. She was referred to our university hospital, and at the time of admission, she was noted to be muted, disheveled, not responding to any commands, and both hands were in flexed posture most of the times. Continuous myoclonic jerks were noted involving all extremities, but predominantly right lower extremity. She was unable to ambulate secondary to worsening ataxia. Her plantar response was extensor in left side and flexor in right. Tone was increased, and brisk reflexes present across all extremities. Jaw jerk was prominent, and bilateral palmomental reflex was present. Laboratory results including hepatitis B, human immunodeficiency virus, syphilis, autoimmune disease workup in addition to thyroid stimulating hormone, vitamin B12, and folate levels were unremarkable. Chest x‐ray, electrocardiogram, and abdomino‐pelvic ultrasound revealed no any abnormalities. Routine CSF results were unremarkable (total counts: 5/mm^3^ with only monocytes, protein: 12mg/dl and sugar: 4.3mmol/l), but 14‐3‐3 protein level was high (4.0 ng/mL). An EEG showed periodic complexes and generalized slowing of wave typical of sporadic CJD (Figure 1). MRI of brain revealed hyperintense signal changes in the bilateral caudate nuclei and the lentiform nucleus with cortical ribboning on diffusion‐weighted image (DWI) in Figure 2, and T2 fluid‐attenuated inversion recovery (FLAIR) sequences in Figure 3. Patient ultimately succumbed to her illness after 1 month of discharge from the hospital which was informed to us in about 1 month postfuneral rituals.

## 3 DISCUSSION

The CJD is an invariably fatal spongiform neurodegenerative disease characterized rapidly progressive dementia, myoclonus, cerebellar, pyramidal, extrapyramidal, visual symptoms, and psychiatric manifestation.^1^, ^2^ CJD occur sporadically in about 85% of cases; 10%‐15% are inherited, <1% are iatrogenic, and <1% are variant.^2^ The commonest, sporadic CJD, also called sCJD, typically presents in the sixth decade, has no predilection for sexes, and has an incidence of approximately one case per million persons per year. sCJD is caused by spontaneous transformation of prion protein or through somatic mutation. Inherited CJD is associated with the mutation in human prion protein gene (*PRNP*). Iatrogenic CJD is extremely rare and, however, has been reported to occur after administration of cadaveric human pituitary hormones, from contaminated neurosurgical instruments, and following corneal or dural graft transplants. Variant CJD is mostly caused by consuming meat from a cow that had bovine spongiform encephalopathy (mad cow disease) and has atypical clinical features, electrophysiological and imaging findings compared to other types.^3^ However, blood or blood product transfusion from asymptomatic vCJD carriers has been associated in few cases as a source of transmission.^5^

The classic triad of sCJD is rapidly progressive dementia, myoclonus, and ataxia. Additional signs include behavioral dysfunction, dysphasia, pyramidal or extrapyramidal signs, cortical blindness, and primitive reflexes.^2^ Most patients decline rapidly to a state of akinetic mutism as in our patient. The mean duration of illness is about 4.5 months, and 80% die within 1 year.^3^ Although the definitive diagnosis of CJD is by neuropathological examination of brain tissue, premortem brain biopsy is not recommended as a routine procedure to confirm the clinical suspicion of sCJD.^6^ According to the Centers for Disease Control and Prevention's criteria for the diagnosis of CJD, our patient fulfills the criteria of the “probable” sporadic CJD.^6^ Our patient exhibited cognitive decline, ataxia, myoclonus, and akinetic mutism. CSF study showed raised protein 14‐3‐3. Her EEG showed longitudinal montage of continuous periodic complexes and generalized slow waves. MRI of brain revealed hyperintense signal changes in the bilateral caudate nuclei and the lentiform nucleus on DWI, T2, and FLAIR images. Furthermore, routine investigations did not indicate an alternative diagnosis. In case of inherited CJD, about 50% of them might not have any history of CJD or neurodegenerative diseases in their family.^7^ In contrast to our case, patient with inherited CJD usually has younger age of onset, slower course, and a longer lifespan than sCJD, though variation in phenotypes has been reported.^8^ However, full sequencing of the *PRNP* gene could not be done in our patient.

Typical EEG occurrences in sCJD are periodic, biphasic, or triphasic sharp‐wave complexes of 1‐2 Hz.^9^ This characteristic EEG pattern is absent in variant CJD.^3^, ^9^ Periodic sharp‐wave complexes (PSWCs) have a sensitivity of 64%‐66% and a specificity of 74%‐91%. However, these characteristic EEG appear only in the advanced stages of the disease.^9^ The characteristic EEG is diagnostic of sCJD in the correct clinical context, and other cases of dementia, such as Alzheimer's disease, Lewy body disease, AIDS dementia, multiple brain abscesses, MELAS syndrome, metabolic, and toxic encephalopathy, rarely have similar EEG findings.^3^

Neuroimaging plays an important role in the diagnosis of sCJD. MRI is the imaging method of choice in sCJD. The role of neuroimaging is to rule out other diseases and look for specific findings of sCJD. The classic MRI manifestations in sCJD are high signals in the basal ganglia or cerebral cortex on DWI, T2, and FLAIR sequences.^9^, ^10^ Despite the high sensitivity and specificity of MRI for sCJD (96% and 93%, respectively), many diseases show similar basal ganglia abnormalities, including Wilson's disease, cerebral hypoxia, and MELAS syndrome, stroke, vasculitis, or reversible posterior leukoencephalopathy.^3^ In some cases of variant CJD, high signals that are confined to the pulvinar on T2 and diffusion‐weighted MRI have been reported, termed as “pulvinar sign”.^10^ However, this pattern has also been reported in few sCJD patients, and could be mistaken for variant CJD.^11^

CSF 14‐3‐3 protein, a marker for neuronal death, detected using Western blotting technology, is a highly sensitive and specific test for diagnosing sCJD. Another importance of this test is that it can be positive even in the early stages of sCJD. However, the CSF14‐3‐3 test in variant CJD is usually negative. Despite its value in the diagnosis of sCJD, many other conditions may also produce 14‐3‐3 protein in CSF, but they can be easily and effectively excluded. These diseases include viral encephalitis, subarachnoid hemorrhage, hypoxic brain damage, metabolic/ toxic encephalopathy, glioblastoma, small cell lung cancer metastasis, paraneoplastic encephalopathy, and corticobasal degeneration.^3^, ^12^

The diagnosis of sCJD can be difficult, and in some cases, auxiliary tests such as EEG, MRI, and CSF studies can be false positives or negatives. However, a new diagnostic method has recently emerged, namely real‐time quacking‐induced conversion (RT‐QuIC) analysis.^13^ This technique takes advantage of the ability of the misfolded pathological prion protein (PrPSc) found in the cerebrospinal fluid, to induce the conversion of normal PrP into a misfolded form. These new misfolded proteins aggregates and are monitored in real time using a fluorescent dye. The CSF RT‐QuIC has sensitivity of 92% and specificity of 100%.^13^, ^14^ With such high sensitivity and specificity, guidelines groups patients with neuropsychiatric disease plus RT‐QuIC positive as probable cases of sCJD, regardless of other clinical features or tests.^6^ Recent studies have compared olfactory epithelial samples obtained from nasal brushes with CSF samples and found that the RT‐QuIC response caused by samples obtained from nasal brushes were stronger and faster than cerebrospinal fluid.^15^, ^16^

In high resource settings, CSF biomarkers like total tau and neurofilament light chain protein(NfL) has been increasingly used in the diagnosis of CJD and other neurodegenerative disorders presenting with dementia. Total tau has been found to have 91% sensitivity and 79% specificity in the diagnosis of CJD which is better than CSF 14‐3‐3 test.^17^ Similarly, high CSF NfL level has been found in CJD in comparison to other neurodegenerative dementias.^18^ Although NfL is the most sensitive (>95%) biomarker for CJD, its specificity is only about 43%.^17^ Thus, CSF total tau is the recommended CSF biomarkers alternative to RT‐QuIC assay for the diagnosis of CJD.

Overall, sCJD is a rapidly developing, fatal neurodegenerative disease that can present in a variety of ways and is difficult to diagnose early. Our case also illustrates the difficulties encountered in sCJD patients when they initially present with psychiatric‐related problems that mask early neurological findings. Since many sCJD patients show psychiatric symptoms early in the disease process, the diagnosis of CJD requires the vigilance of primary care physicians and psychiatrists.^9^, ^19^ A timely meeting with a neurologist will undoubtedly help with accurate diagnosis and prognosis. There are currently no disease‐modifying therapies for sCJD, but rapid diagnosis can initiate appropriate palliative care and provide some emotional support both for patient and family members. Current treatment focus to relieve symptoms and make people as comfortable as possible. Opioids can help alleviate pain if it occurs, while clonazepam and sodium valproate can help relieve myoclonus. At the end of the disease, tube feeding or total parenteral nutrition may be required.^20^

Prompt diagnosis can also help reduce the risk of iatrogenic transmission. sCJD has been transmitted through contaminated human pituitary‐derived growth hormone, dura mater grafts, corneal transplant, brain electrodes, and neurosurgical instruments.^4^, ^21^, ^22^ There are also cases of blood‐mediated transmission, but this only applies to individuals with variant CJD, not to sCJD individuals.^4^, ^23^ During neurosurgery or brain biopsy, surgeons who deal with brain tissue may pose a lower risk of self‐inoculation.^3^, ^4^, ^21^ Studies have also found that there is an ample prion activity in the nasal vault, and viral seeding of the nasal vault may occur, leading to transmission.^15^, ^16^ To reduce the risk of sCJD transmission from one person to another, people who suspected or are diagnosed with CJD should not donate blood, tissues, or organs. Instruments used for exploration, diagnosis, and treatment of nasal cavity or brain of sCJD patient should be sterilized with specific procedures or discarded. After death, forensic pathologists or those who handle the body during the funeral must remain vigilant and do not expose themselves to tissues or fluids known to harbor prion.^4^, ^24^

## 4 CONCLUSION

sCJD can present in a variety of ways and can be difficult to diagnose early, especially if they initially show up with a psychiatric‐related problem that obscures neurological findings. The diagnosis of CJD requires the vigilance of primary care physicians and psychiatrists, and timely referral to neurologists is undoubtedly helpful for accurate diagnosis and prognosis. Although sCJD is always fatal and no accepted treatment is currently available, it is important to make an early and accurate diagnosis so that the prognosis and plan of proper management could be explained to caretakers and family members.

## CONFLICT OF INTEREST

None declared.

## AUTHORS CONTRIBUTIONS

RO: involved in writing the manuscript, concept, collection of case information, manuscript revision. GN and SJ: participated in preparing literature review and interpretation of clinical findings. BPG, RK, and RR: involved in patient care team and collection of case information. All authors approved the final version.
